# Immunotherapy Use Prior to Liver Transplant in Patients with Hepatocellular Carcinoma

**DOI:** 10.3390/curroncol29120771

**Published:** 2022-12-13

**Authors:** Stephanie M. Woo, Alexandra V. Kimchy, Lynette M. Sequeira, Charles S. Dorris, Aiwu R. He, Amol S. Rangnekar

**Affiliations:** 1 Department of Gastroenterology and Hepatology, MedStar Georgetown University Hospital, 3800 Reservoir Road NW, Washington, DC 20007, USA; 2 Department of Internal Medicine, MedStar Georgetown University Hospital, Washington, DC 20007, USA; 3 Georgetown University School of Medicine, Washington, DC 20007, USA; 4 Dahlgren Memorial Library, Georgetown University Medical Center, Washington, DC 20007, USA; 5 Department of Hematology and Oncology, MedStar Georgetown University Hospital, Washington, DC 20007, USA; 6 MedStar Georgetown Transplant Institute, MedStar Georgetown University Hospital, Washington, DC 20007, USA

**Keywords:** hepatocellular carcinoma, immunotherapy, liver transplant, immunosuppression, liver cancer

## Abstract

Hepatocellular carcinoma (HCC) is the fourth leading cause of cancer-related mortality worldwide, and its incidence has increased rapidly in the United States over the past two decades. Liver transplant is considered curative, but is not always possible, and pre-transplant immunotherapy is of great interest as a modality for downstaging the tumor burden. We present a review of the literature on pre-liver transplant immunotherapy use in patients with HCC. Our literature search queried publications in Ovid MEDLINE, Ovid Embase, and Web of Science, and ultimately identified 24 original research publications to be included for analysis. We found that the role of PD-1 and PD-L1 in risk stratification for rejection is of special interest to researchers, and ongoing randomized clinical trials PLENTY and Dulect 2020-1 will provide insight into the role of PD-1 and PD-L1 in liver transplant management in the future. This literature search and the resulting review represents the most thorough collection, analysis, and presentation of the literature on the subject to date.

## 1. Introduction

Since the advent of immune checkpoint inhibitors, ushered in by the FDA-approval of CTLA-4 inhibitor ipilimumab in the treatment of melanoma in 2011, immunotherapy has gained increasing popularity in the treatment of a variety of cancers [1,2,3]. For unresectable hepatocellular carcinoma, treatment options depend on tumor burden and severity of liver disease, but liver transplant (LT) is generally considered to be curative [4]. Prior to LT, locoregional strategies are commonly utilized to reduce tumor burden and optimize patients’ tumor profiles to meet or maintain criteria for transplantation [5]. Interest in immunotherapy as bridging therapy has grown, and while most data are derived from case reports and limited case series, early findings are promising [6].

Systemic immunotherapy is an approved therapy for extensive HCC in patients who are not transplant candidates [5]. In fact, recent clinical trials and Food and Drug Administration guidelines have validated and established various immunotherapy regimens as both first and second-line therapies for unresectable HCC [7,8,9]. Nevertheless, the use of immunotherapy has historically been discouraged in patients with preexisting organ transplantation due to the risk for graft rejection [10]. Thus, we set out to evaluate if immunotherapy is a safe and effective option to use in patients with extensive hepatic tumors prior to liver transplant. We also aim to elucidate the necessary time before liver transplant that immunotherapy should be administered to avoid graft rejection.

Here, we present a robust discussion of the available literature surrounding the use of pre-liver transplant immunotherapy, representing to the best of our knowledge the most thorough literature review on the topic at the time of this publication.

## 2. Hepatocellular Carcinoma Therapy

### 2.1. Early Stage HCC Standard of Care

Tumor staging plays a major role in determining the treatment strategy with the greatest predicted benefit. The current recommendation is for patients with early stage disease (BCLC 0-A) to pursue curative therapies such as local resection, ablation or LT given favorable outcomes with a 70–80 percent 5-year survival rate. The decision to proceed with resection of HCC is multifactorial and includes assessment of liver function, performance status, portal hypertension and tumor characteristics. Resection is the first-line treatment option for HCC in patients without cirrhosis. In early stage HCC, resection may also be considered for patients with cirrhosis without portal hypertension. Local ablation is another curative strategy that can be considered for patients with BCLC 0 or A stage disease who are not suitable for surgery [11,12]. LT is the recommended treatment for BCLC stage A tumors that are within Milan criteria (single tumor less than or equal to 5 cm or 2–3 tumors less than or equal to 3 cm without vascular invasion) [11]. It is a highly effective option for HCC as it not only removes the tumor burden but also treats the underlying liver disease [13]. The outcomes reported after LT are favorable, with a 5-year survival rate of approximately 70% and recurrence rate of between 10 to 15 percent [11]. Downstaging may allow patients with more advanced intrahepatic tumor burden to become candidates for LT. The management of HCC differs in patients with intermediate and advanced stage disease often requiring alternative therapeutic options such as local regional or systemic therapies [11].

### 2.2. Bridging Therapies

Although LT is a more definitive treatment strategy in terms of removing the primary tumor and reducing the risk of future malignancy, it is not an immediate option for all patients given the limited supply of donor livers and selection requirements that must be met to be listed. According to AASLD guidelines, patients beyond Milan criteria should be considered for liver transplant after downstaging therapy to meet the criteria [13]. Bridging therapies are recommended for patients who spend prolonged periods on the waitlist and to decrease progression of disease [14]. The AASLD does not favor one bridging strategy over another in patients awaiting transplant who are within Milan criteria [13].

### 2.3. Procedural Interventions

Local regional therapy is the most recommended choice of treatment for unresectable BCLC stage A and B disease. Radiofrequency ablation is indicated for smaller lesions less than 3 cm and has similar survival outcomes as surgical resection in lesions less than 2 cm [15]. Other ablative techniques including microwave and cryoablation as well as stereotactic body radiation therapy may be considered for larger tumors. In patients with stage B disease, transarterial chemotherapy has become the standard treatment used given the survival benefit reported by randomized controlled trials [15]. The use of drug eluting beads offers more consistent dosing and decreased systemic absorption over conventional transarterial chemotherapy [13]. Transarterial radioembolization using microspheres coated in radioactive isotopes to embolize tumor feeding vessels while maintaining patency of the hepatic artery has emerged as an alternative to transarterial chemotherapy [13,15]. As direct comparison studies are limited, there are not well-defined recommendations for one of the two treatment modalities. Transarterial radioembolization has been used in large tumors with portal vein invasion or progression of disease after transarterial chemotherapy [13]. The use of combination therapy with local regional therapy and systemic therapy was initially not advisable due to the lack of clinical efficacy in initial studies of transarterial chemotherapy with targeted therapy [13]. However, there has been further investigation of the timing of combination therapy with the TACTICS trial demonstrating survival benefit when sorafenib preceded the use of TACE [15]. With the development of immunotherapies for HCC, there are ongoing trials evaluating the utility of various combinations of locoregional therapy with immunotherapy and immunotherapy plus targeted therapy [15].

### 2.4. Targeted Therapies

Systemic therapy is recommended for patients with BCLC stage C as well as stage B disease that has progressed after local regional therapy. Sorafenib, an oral tyrosine kinase inhibitor, has been considered a first-line therapy for advanced HCC since the SHARP trial demonstrated a survival benefit in 2007, which was confirmed in the Asia-Pacific trial [16]. Since then, there have been multiple agents that have been studied and approved for use as second-line therapy for advanced HCC. In 2018, another oral tyrosine kinase inhibitor, lenvatinib, was approved as a first-line therapy after the REFLECT trial showed non-inferiority to sorafenib along with a better tolerability profile [16]. Regorafenib, a tyrosine kinase inhibitor, and cabozantinib, a combination tyrosine kinase and c-MET inhibitor, have both been studied in patients who have had disease progression on sorafenib and are approved as second-line therapies [16]. The side effects that are typical for the tyrosine kinase inhibitors include hypertension, hand-foot skin reaction, fatigue and diarrhea [13]. In the 2019 REACH trial, the recombinant anti-vascular endothelial growth factor receptor 2 monoclonal human antibody, ramucirumab, showed a survival benefit in patients previously treated with sorafenib with an alpha fetoprotein level greater than or equal to 400 ng/mL and was approved as a second-line therapy for this subset of patients. Common side effects include HTN, fatigue, peripheral edema and ascites [16].

### 2.5. Immunotherapies

The growing disease burden of HCC worldwide has resulted in an increase in studies investigating the efficacy of new systemic therapies, particularly immunotherapeutic agents, given the clinical utility of these drugs in other cancer types [16,17]. Nivolumab, a PD-1 immune checkpoint inhibitor, was initially used in patients with advanced tumors resistant to standard therapies including melanoma, renal cell carcinoma, and non-small-cell carcinoma [17]. It later became the first immunotherapy approved for advanced HCC in 2017. The CheckMate 040 and 459 trials demonstrated a survival benefit of nivolumab in treatment naive or previously treated patients with sorafenib, although it was not superior to the first-line therapy, sorafenib [16]. A second PD-1 antibody, pembrolizumab, was then approved as a second-line therapy in 2018 after the Keynote 224 trial showed positive results in patients with previously treated advanced HCC [16,18]. In 2020, the CheckMate 040 study showed that nivolumab in combination with ipilimumab, a CTLA-4 immune checkpoint inhibitor, had an overall response rate of 33% in previously treated patients with advanced HCC and was approved as a second-line therapy [16]. Shortly thereafter the combination therapy, atezolizumab, a PDL-1 inhibitor, with bevacizumab, a vascular endothelial growth factor inhibitor, demonstrated a significant survival benefit over the standard therapy, sorafenib, in the IMBrave 150 trial. This combination then received approval as a first-line therapy for unresectable HCC based on the results of this phase 3 trial [16].

There is significant concern regarding the safety of using immunotherapy in patients with HCC who may later undergo liver transplant, especially given the risk of immune-related adverse events. The most frequently affected organs by immune-related adverse effects include the liver, colon, lung, skin and endocrine tissue [19]. Immune-related adverse events occur in 60–85% of patients treated with the anti-CTLA4 agent, ipilimumab, at a dose of 3 mg/kg with 10–27% experiencing grade 3 to 4 toxicities. Rash and colitis typically develop first around weeks 4–6 after initiation of treatment followed by liver toxicity around weeks 6–8 [19]. High grade adverse events are less frequent with anti-PD-1 therapies, nivolumab and pembrolizumab, than observed with ipilimumab. The most common reported adverse effect with anti-PD-1 agents is fatigue [19]. In the IMBrave 150 trial, grade 3 to 4 toxicities were reported in 38% of patients receiving combination therapy with atezolizumab and bevacizumab. The most frequent events were hypertension, fatigue and proteinuria. Bevacizumab has also demonstrated an increased risk of bleeding [16]. Hepatotoxicity occurs in approximately 5–10% of patients treated with nivolumab, pembrolizumab or ipilimumab monotherapy, and increases to 25–30% in patients treated with combination therapy of nivolumab and ipilimumab [19]. Immune-related hepatitis tends to present as an asymptomatic elevation in alanine or aspartate aminotransferase with or without elevation in bilirubin. Liver biopsy findings have been consistent with a predominant hepatocyte injury pattern with sinusoidal histiocytic infiltrates, central hepatic vein damage and endothelial inflammation like autoimmune hepatitis, or a predominant bile-duct injury pattern [20]. Overall immune-related adverse events typically occur early after initiation of immunotherapy; however, there have been instances of delayed toxicity after discontinuation of treatment [19]. This delayed effect is thought in part to be due to drug pharmacodynamics with immunotherapeutic agents having a longer duration of effect. Anti-PD1/PD-L1 agents such as nivolumab, pembrolizumab, and atezolizumab have a half-life of approximately 4 weeks while the anti-CTLA4 agent, ipilimumab, has a half-life of about 2 weeks [17,19]. The possibility of delayed effects of neoadjuvant immunotherapy is an important consideration when assessing transplant candidacy [19]. See Table 1. “Examples of FDA-approved immunotherapies and their half-lives”.

## 3. Pre-Transplant Use of Immunotherapy

### 3.1. Patient Demographics

In our literature search, we found 24 original research publications that focused on pre-liver transplant immunotherapy use in HCC patients and included all of them for our analysis. We identified 45 patients who received immunotherapy prior to liver transplant. Of these patients, 67% were male (30 of 45), 16% were female (7 of 45), and the remaining patients did not specify. The mean age of the patients included was 57 years (min 14 years, max 68 years).

All the patients included received immunotherapy for hepatocellular carcinoma as their primary malignancy. HCV was the leading etiology for primary underlying disorder in 29% of patients (13 of 45), followed by HBV (10 of 45), and alcohol (3 of 45). Of note, two patients had overlapping HCV and alcoholic liver disease. Cirrhosis was definitively reported in 24% of patients (11 of 45). Milan criteria was reported for 21 patients, of whom 16 were within Milan criteria. Of the two patients that were outside of Milan criteria, one eventually was downstaged to within criteria after therapy. See Table 2. “Cases of immune checkpoint inhibitors use in the pre liver transplant setting”.

### 3.2. Immunotherapy Used

A total of 42 of 45 patients were treated with PD-1 inhibitors, 3 of 45 patients were treated with PD-L1 inhibitors, and no patients were treated with CTLA4 inhibitors. The most used PD-1 inhibitor was nivolumab, comprising 33 of 45 immunotherapy modalities. Duration of therapy was quantified differently across studies, as some authors reported number of cycles while others reported months, making it difficult to compare. Of the 12 studies that reported treatment duration in cycles, the average was 9.17 cycles (min 1, max 34). A variety of other pre-treatment modalities were reported, the most common of which was TACE, followed by microwave ablation and resection.

### 3.3. Timing of Immunotherapy

The timing between the last dose of immunotherapy and liver transplant is an important factor to explore in terms of post-operative outcomes, with the literature suggesting a waiting period of 4–8 weeks [37]. As mentioned previously, the half-life of immunotherapeutic agents can be taken into consideration when determining the optimal timing of transplant. There is concern that the effects of immunotherapy may be longer in duration than anticipated by the drug’s pharmacodynamic profile given instances of delayed toxicity after discontinuation of treatment described in adverse event reports [19]. In our literature search, we found that in patients treated with nivolumab who received a liver transplant within one half-life (27 days) of the drug experienced acute rejection in three out of four (75%) of cases. Nordness et al. reported a case of a 65-year-old male with HCV cirrhosis who received a liver transplant 8 days following his last cycle of nivolumab and experienced acute rejection that was managed with rATG and solumedrol but subsequently had worsening allograft function and care was withdrawn [29]. In another case reported by Schnickel et al., the patient experienced acute rejection after receiving a liver transplant for HCV cirrhosis who had received treatment with nivolumab 10 days prior. However, the graft was able to be salvaged after receiving rATG, steroid pulse, rituximab and IVIG [32]. Aby et al. described a case of a 64-year-old male with HCV cirrhosis who was treated with nivolumab 16 days prior to liver transplant and experienced acute rejection that resolved with treatment with solumedrol and thymoglobulin [22]. There was one case reported by Chen et al. in which a patient received a liver transplant 7 days after treatment with nivolumab who did not experience acute rejection [24]. It is notable that this patient received only one cycle of nivolumab for his total treatment course prior to transplant whereas the other patients who experienced acute rejection received extended courses of treatment ranging from 8 months to 2 years [22,24,29,32].

In our review, the two longest observed intervals between last immunotherapy use and transplant exceeded 5 half-lives of nivolumab (135 days, 97% clearance), and these patients did not experience acute rejection. Interestingly, we observed one patient with an interval of 4.9 half-lives who experienced rejection. All other patients who experienced rejection had an interval time of less than 3.5 half-lives between their last dose of immunotherapy and transplant. Peterson et al. reported a patient with HCV cirrhosis who was successfully transplanted 304 days after treatment with nivolumab [30]. Another patient with HCV cirrhosis described by Schnickel et al. was transplanted 6 months following treatment with nivolumab and had no evidence of rejection [32]. As plasma drug concentrations typically decline below clinically significant levels after 3 half-lives, we compared the rates of acute graft rejection among patients transplanted before and after 3 half-lives of nivolumab [21]. There were 6 cases of acute rejection out of 19 patients (32%) who had received a liver transplant within 3 half-lives of nivolumab treatment. Acute rejection was present in 2 out of 14 patients (14%) who were transplanted beyond 3 half-lives of nivolumab treatment. Based on our results, the half-life of the immunotherapeutic agent used may be considered when timing liver transplant following treatment; however, additional cases are needed to further elucidate a minimum safe interval between transplant and immunotherapy use.

### 3.4. Graft Rejection

#### 3.4.1. Timing of Rejection

Among the 11 patients in our review who developed rejection, the majority experienced graft rejection between 6 to 14 days post-transplant. These patients were either treated with pembrolizumab or nivolumab monotherapy or in combination with a tyrosine kinase inhibitor. There were two patients who had rejection within 48 h of transplant and with varying latency time from last immunotherapy dose. One patient described by Chen et al. was treated with toripalimab in combination with lenvatinib 93 days prior to transplant [24]. He developed fatal acute hepatic necrosis 33 h after transplant that did not respond to treatment with continuous renal replacement therapy, plasma exchange and plasma specific bilirubin absorption [24]. The other patient reported by Yin et al. had received atezolizumab in combination with lenvatinib 19 weeks prior to transplant and experienced fatal acute graft rejection 20 h after transplant [36]. Our results indicate that the timing of rejection post-transplant may be related to the specific immunotherapeutic agent used. Among the cases in this review, there was no relationship observed between the time interval of immunotherapy used prior to liver transplant and time to rejection following transplant.

#### 3.4.2. Role of PD1/PD-L1 Expression in Rejection

There is evidence to suggest that PD-L1 expression in the allograft tissue may play a role in the development of donor graft failure. Chen, G et al. described a case of acute graft rejection in a patient who received treatment with the anti-PD-1 antibody toripalimab pre-transplant. The pre-implant donor liver tissue was negative for PD-L1 expression, but the post-implant tissue was positive [24]. The authors proposed that toripalimab may have led to the donor graft’s inability to evade the host immunologic response due to the expression of PD-L1 [24]. In a case reported by Deghan et al., the patient received treatment with nivolumab prior to liver transplant and later developed acute graft rejection following transplant. The tissue biopsy from the allograft showed rare staining of PD-L1 with intermediate staining of PD-1 [26]. Nordness et al. described another patient who experienced acute graft rejection and received treatment with nivolumab prior to transplant [29]. In this case, the pre-implant liver tissue was negative for PD-L1 expression whereas the postoperative day 6 liver biopsy was positive. Although the authors agreed that PD-L1 expression may be a marker of subclinical rejection, they proposed that upregulation of PD-L1 in the allograft may have a protective effect against an immunologic response thereby preventing early rejection [29]. These studies demonstrate a possible relationship between PD-1/PD-L1 expression in donor liver grafts and acute graft rejection; however, the mechanism of rejection with immune-checkpoint inhibitor use requires further investigation.

#### 3.4.3. Treatment of Rejection and Outcomes

Our review revealed variation in treatment and outcomes of allograft rejection in patients who had received immunotherapy during the pre-transplant period. A total of 11 patients were found to have rejection, 2 of which were antibody mediated, while the remainder were either cellular or unspecified. Of the eight cases with known treatment of acute graft rejection, there were four cases that resulted in resolution of rejection. Steroids were used as the primary treatment modality in three of the four cases with concomitant use of anti-thymocyte globulin in two cases. In a case reported by Schnickel et al., the patient additionally received rituximab, an anti-CD20 antibody, and IV immunoglobulin for moderate rejection, which resulted in salvage of the donor liver graft [32]. Tabrizian et al. described a case of mild acute rejection that responded to increased dosage of tacrolimus, a calcineurin inhibitor [35]. Among the fourcases that resulted in graft loss, concomitant use of steroids and anti-thymocyte globulin were used in three of the cases. Gao et al. proposed plasmapheresis as a potential treatment modality to wash out remaining effects of immunotherapy agents given the reversibility of target-binding [14]. In three of the four cases, plasma exchange was employed in addition to these medical therapies along with the implementation of IV Immunoglobulin therapy in two cases. However, none of the grafts were able to be salvaged. Chen et al. described a case in which the patient underwent plasma exchange with a bilirubin specific absorption column, but this still resulted in fatal graft failure [23]. There were two cases of graft failure that resulted in death and two cases where the patients received a new liver transplant. Finally, treatment efforts in the two patients who demonstrated antibody-mediated rejection resulted in death in one and graft loss with successful re-transplant in the other. While antibody-mediated rejection is rare, we believe it may be more difficult to treat. Given the differing results with similar immunosuppressive strategies, there may be other factors that play a larger role in the outcome of acute graft rejection following transplant. However, it is notable that the timing of liver transplant following immunotherapy use did not correlate with the outcome of graft rejection in the cases included in our review.

#### 3.4.4. Induction and Maintenance Immunosuppression

The role of induction and maintenance therapies in patients who have received immunotherapy prior to liver transplant is an area that requires further exploration. Immunosuppression is crucial for graft protection; however, it may also negate the antitumor effects of immunotherapy. As such, determining the ideal immunosuppression regimen and schedule is crucial. Katariya et al. suggested that T-cell depleting agents may offer some protection from an immune response against the donor graft if there is persistent immunotherapy activity following discontinuation of treatment [17]. In our review, there were six cases that received induction with anti-thymoglobulin monotherapy in which two patients (33%) experienced acute rejection following transplant. There was one case that received a combination of anti-thymoglobulin and steroids for induction, which resulted in a successful transplant. Another common combination therapy used for induction was steroids, mycophenolate, an antimetabolite, and tacrolimus, and 2 out of 10 cases (20%) experienced acute allograft rejection. Qiao et al. reported one case of acute graft rejection among seven patients (14%) who had received induction therapy with basiliximab, a chimeric monoclonal anti-interleukin 2 receptor antibody [31]. However, there were 15 cases included in our review in which no induction therapy was used prior to transplant and only 2 patients (13%) developed allograft rejection. Based on these results, the utility of induction therapy in patients who have received immunotherapy treatment prior to liver transplant remains uncertain and is an area in need of further investigation.

The concomitant use of immunotherapy and maintenance immunosuppression post-transplant in patients has been an area of concern due to their opposing mechanisms of action [22]. However, the effects of pre-transplant immunotherapy use on post-transplant immunosuppression remains uncertain. In our review, the most common maintenance immunosuppressive regimen used was a combination of tacrolimus, mycophenolate and steroids. Of the 18 patients who received this regimen, 6 patients (33%) experienced acute graft rejection. Qiao et al. reported one case of acute rejection in seven patients (14%) who received combination maintenance therapy with a calcineurin inhibitor (cyclosporine or tacrolimus), sirolimus, a mammalian target of rapamycin inhibitor, mycophenolate, and steroids [31]. The one patient that received a combination of tacrolimus and steroid therapy had acute graft rejection following transplant. There were six cases in our review that received a combination of tacrolimus and mycophenolate for maintenance immunosuppression and no patients experienced allograft rejection. In addition, there was one patient that received a combination of tacrolimus and sirolimus who did not experience rejection. Overall, further cases are needed to determine the optimal maintenance immunosuppressive regimen in patients who received pre-transplant immunotherapy treatment (See Table 2: “Cases of immune checkpoint inhibitors use in the pre liver transplant setting”).

## 4. Current Trials Examining Pre-LT Immunotherapy Use

At present, there are two trials, both based at RenJi Hospital in Shanghai, China, enrolling patients to prospectively investigate the impact of pre-liver transplant immunotherapy use. The PLENTY trial (NCT04425226) is an open label randomized clinical trial examining the safety and efficacy of pembrolizumab with neoadjuvant Lenvatinib in the treatment of HCC prior to liver transplant. It was launched in 2020 and has recruited 192 patients to date, with estimated completion in 2024. The primary outcome measure is recurrence-free survival with secondary outcome measures including disease control rate, percentage of patients who experience adverse events, and objective response rate. Preliminary data from the PLENTY trial were recently published in an abstract, stating that early results are promising. Specifically, outcomes that are being investigated include recurrence-free survival and objective response rate [38].

Meanwhile, the open label Dulect2020-1 (NCT04443322) trial investigates the safety and efficacy of durvalumab with Lenvatinib in patients with HCC before liver transplant and metastatic unresectable HCC, with two primary outcome measures: progression free survival and recurrence-free survival. Secondary outcome measures include objective response rate, overall survival, and percentage of patients who experience an adverse event. So far, 20 patients have been enrolled in the Dulect2020-1 trial, with the enrollment period starting in 2020 and anticipated in end by 2025.

## 5. Post-Liver Transplant Immunotherapy Use

While an extensive exploration of post-transplant immunotherapy use is beyond the scope of this review, the following discussion highlights the field’s main developments and future directions for its study.

The risk for HCC recurrence after liver transplant is 15% to 20% and confers a poor prognosis with median survival ranging from 7 to 16 months [39,40]. Treatment options for recurrent HCC following liver transplant are the similar as those available in the pre-transplant setting. Potential treatments include locoregional therapies and surgical resection for local recurrence. However, over 50% of HCC recurrence involves other organs, warranting systemic therapies including Sorafenib, regorafenib, and traditional chemotherapy [41,42].

Furthermore, post-LT patients remain at risk for developing other de novo malignancies, including melanoma and NSLC, which are treated with immunotherapy. In fact, according to data from the United States Scientific Registry of Transplant Recipients database, the estimated incidence of de novo extrahepatic malignancy is 1.3% in the first year post-LT and increases to 18.8% after twenty years [42].

While immunotherapy for recurrent HCC has been explored, there is a risk for graft rejection, and there are no guidelines regarding its use [31]. Immunotherapy use in the post-transplant setting remains controversial and is not routinely considered at our institution. In a recent systematic review by Yin et al., of 28 patients who received immunotherapy post-LT, 32% (9 of 28) experienced biopsy-proven acute graft rejection. Of the 9 patients with acute rejection, all were treated with immunosuppression, 44% (4 of 9) cases resolved, 33% (3 of 9) cases progressed to graft failure leading to death, and one had an unknown response [36]. Interestingly, Yin et al. note that the three cases of graft failure were in patients under 60 years of age, raising the concern for a stronger immune response in the younger population, increasing their risk for rejection.

Yin et al. found that patients who developed rejection experienced a shorter period between LT and immunotherapy compared to those who did not [36]. Anugwom et al. found that liver rejection to be virtually nonexistent among patients who receive immunotherapy two to eight years after transplant, hypothesized to be from an increased immunological tolerance for the graft over time [42]. However, in their systematic review of immune-checkpoint inhibitors following transplant of various solid organs, including liver, kidney, and heart, Kumar et al. did not find a significant association between rejection and time since transplant to initiation of ICI [43]. Further study on timing of immunotherapy initiation following transplant is warranted.

The appropriate methods for immunosuppression remain unknown. Kumar et al. did find a significant association between type of immunosuppression and rejection, where patients treated with only low dose prednisone saw higher rates of rejection than those who received tacrolimus or other combination immunosuppression regimens [43].

Furthermore, studies have shown an association between acute rejection and the presence of PD-L1 positivity [36,44]. Yin et al. found that of the four patients whose biopsies expressed PD-L1 positivity, all developed acute rejection [36]. Better understanding of this relationship may help guide treatment in the future.

## 6. Conclusions

Immunotherapy is a promising method of downstaging the hepatocellular carcinoma burden. It is possible that some patients with incomplete response to immunotherapy and absence of extrahepatic HCC may benefit from subsequent liver transplantation. However, the question of whether liver transplant confers additional survival benefit in patients with complete response to immunotherapy needs to be elucidated. While the question of timing remains unanswered, our literature suggests that the optimal waiting time between immunotherapy, specifically PD-1 and PD-L1 inhibitors, and liver transplant is at least eight weeks, or two half-lives. The results from the prospective PLENTY and Dulect 2020-1 trials remain highly anticipated and will likely guide studies in the future. Similarly, the optimal immunosuppression regimen remains unknown, though our results show suggest that induction immunosuppression may be unnecessary. One of the most interesting clinical questions is the role of biomarkers. The literature has shown that PD-1 and PD-L1 expression has the potential to enhance risk stratification, prevention, and treatment of rejection. While it is too early to make definitive claims on the safety and efficacy of immunotherapy as downstaging modality for HCC prior to LT, we believe that at this time, the potential benefits outweigh the risks and further studies are warranted.

## Figures and Tables

**Table 1:** Examples of FDA-approved immunotherapies and their half-lives.

	Trade Name	Mechanism	Half-Life
Nivolumab	Opdivo	PD-1 Inhibitor	26.7 days (FDA 2014)
Pembrolizumab	Keytruda	PD-1 Inhibitor	23 days (FDA 2016)
Atezolizumab	Tecentriq	PD-L1 Inhibitor	27 days (FDA 2018)
Durvalumab	Imfinzi	PD-L1 Inhibitor	18 days (FDA 2018)
Ipilimumab	Yervoy	CTLA-4 Inhibitor	15.4 days (FDA 2015)

**Table 2:** Cases of immune checkpoint inhibitors use in the pre-liver transplant setting.

Author	Age (Years)	Underlying Liver Disease	Graft PD1 Status	ICI Therapies Used	Duration of Therapy	Other Pre-Interventions	Time from Last Dose ICI to LT	Rejection	Time to Rejection	Treatment of Rejection	Outcome
Abdelrahim et al. [21]	66	HCV; cirrhosis	Unknown	Atezolizumab Bevacizumab	6 cycles 5 cycles	None	2 months	None	NA	NA	No recurrence or rejection
Aby et al. [22]	64	HCV; cirrhosis	Unknown	Nivolumab	23 cycles	Radio- and chemo- embolization, microwave ablation; sorafenib	16 days	Moderate to severe acute cellular rejection	9 days	Solumedrol, thymoglobulin	Resolved rejection
Chen, G et al. [23]	39	HBV; unknown cirrhosis status	Positive	Toripalimab Lenvatinib	10 cycles Unknown	Resection, TACE, RFA, sorafenib, microwave ablation during ICI	93 days	Acute hepatic necrosis, antibody mediated	33 h	Plasma exchange, plasma-specific bilirubin adsorption, CRRT	Death
Chen, Z. et al. [24]	64	Cirrhosis of unknown etiology	Unknown	Nivolumab	1 cycle	None	7 days	NA	NA	NA	Recurrence without rejection
Chen, Z. et al. [24]	47	Cirrhosis of unknown etiology	Unknown	Nivolumab	1 cycle	TACE	122 days	NA	NA	NA	Recurrence without rejection
Chen, Z. et al. [24]	50	Cirrhosis of unknown etiology	Unknown	Nivolumab	1 cycle	TACE	62 days	NA	NA	NA	No recurrence or rejection
Chen, Z. et al. [24]	38	Cirrhosis of unknown etiology	Unknown	Nivolumab	6 cycles	TACE, RFA	59 days	NA	NA	NA	No recurrence or rejection
Chen, Z. et al. [24]	67	Cirrhosis of unknown etiology	Unknown	Nivolumab	6 cycles	TACE	67 days	NA	NA	NA	No recurrence or rejection
Dave et al. [25]	63 (average)	2 HCV 2 HCV and alcoholic 1 HBV 1 NASH All with unknown cirrhosis status	Unknown	Nivolumab	Unknown	2 received loco-regional therapy	105 days (average)	2 rejections, non-specific immune-mediated	Unknown	Unknown	2 graft losses, with 1 re-transplant successful
Dehghan et al. [26]	60	HCV; cirrhosis	PDL1 rare, PD1 intermediate	Nivolumab	16 cycles 3 months	TACE, microwave ablation (repeat ablation during ICI), sorafenib	5 weeks	Acute cellular and antibody mediated rejection with submassive hepatic necrosis, with CD3 lymphocytes and DSA	10 days	Methyl-prednisolone, anti-thymocyte globulin, IVIG, plasma exchange	Graft loss, but re-transplant was successful
Kang et al. [27]	14	Unknown	Unknown	Pembrolizumab	3 cycles	Cisplatin, Doxorubicin, Dexrazoxane, TACE, Tri-segmentectomy	138 days	None	NA	NA	No recurrence or rejection
Lizaola et al. [28]	63	NASH; cirrhosis	Unknown	Nivolumab Ipilimumab	6 months	Radio-embolization, sorafenib	8 weeks	None	NA	NA	No recurrence or rejection
Nordness et al. [29]	65	HCV; unknown cirrhosis status	Positive	Nivolumab	2 years	Resection, radio-embolization, sorafenib, TACE during ICI	8 days	Acute rejection with hepatic necrosis and lymphocytic infiltration	6 days	Methyl-prednisolone, anti-thymocyte globulin	Death
Peterson et al. [30]	68	HCV; cirrhosis	Unknown	Nivolumab	Unknown	Radio-embolization	10 months	None	NA	NA	No recurrence or rejection
Qiao et al. [31]	53 (average)	Unknown	Unknown	Pembrolizumab Lenvatinib	1–5 cycles	None	1.3 months	Acute cellular rejection in 1 patient, T-cell mediated	10 days	Methyl-prednisolone	Resolved
Schnickel et al. [32]	61	HCV; unknown cirrhosis status	Unknown	Nivolumab	18 months	None	5 weeks	Acute cellular rejection (with 60% necrosis) with DSA	12 days	RATG, steroid pulse, plasma-pheresis, IVIG	Graft failure with re-transplant
Schnickel et al. [32]	65	HCV; unknown cirrhosis status	Unknown	Nivolumab	8 months	None	10 days	Acute cellular rejection	14 days	RATG, steroid pulse, rituximab, IVIG	Salvaged graft
Schnickel et al. [32]	71	HBV; unknown cirrhosis status	Unknown	Nivolumab	8 months	None	83 months	None	NA	NA	No recurrence or rejection
Schnickel et al. [32]	65	HCV; unknown cirrhosis status	Unknown	Nivolumab	12 months	None	4 months	None	NA	NA	No recurrence or rejection
Schnickel et al. [32]	68	HCV; unknown cirrhosis status	Unknown	Nivolumab	12 months	None	6 months	None	NA	NA	No recurrence or rejection
Schwacha et al. [33]	62	Alcoholic cirrhosis	Unknown	Nivolumab	34 cycles	Sorafenib, Regorafenib, Microwave ablation	21 weeks	None	NA	NA	No recurrence or rejection
Sogbe et al. [34]	61	HBV with unknown cirrhosis status	Unknown	Durvalumab	15 months	Sorafenib	90 days	None	NA	NA	No recurrence or rejection
Tabrizian et al. [35]	57	5 patients with HBV; 4 patients unknown, all with unknown cirrhosis status	Unknown	Nivolumab	2–32 cycles (median: 9 cycles)	Locoregional therapy (including chemo- and radio-embolization, ablation, radiation)	Within 4 weeks	Mild rejection in 1 patient due to low tacrolimus levels	Unknown	Increased tacrolimus dosage	Resolved
Yin et al. [36]	37	HBV with unknown cirrhosis status	Unknown	Atezolizumab Lenvatinib	6 months	TACE, Microwave ablation	None	Severe non-specific immune-mediated rejection	Unknown	Unknown	Death

HCC: Hepatocellular carcinoma; HBV: hepatitis B virus; HCV: hepatitis C virus; TACE: trans arterial chemoembolization; RFA: radiofrequency embolization; IVIG: intravenous immune globulin; DSA: donor-specific antibody; RATG: rabbit anti-thymocyte globulin.
